# Active learning with label quality control

**DOI:** 10.7717/peerj-cs.1480

**Published:** 2023-09-08

**Authors:** Xingyu Wang, Xurong Chi, Yanzhi Song, Zhouwang Yang

**Affiliations:** University of Science and Technology of China, Hefei, China

**Keywords:** Active learning, Automated optical inspection, Label quality control

## Abstract

Training deep neural networks requires a large number of labeled samples, which are typically provided by crowdsourced workers or professionals at a high cost. To obtain qualified labels, samples need to be relabeled for inspection to control the quality of the labels, which further increases the cost. Active learning methods aim to select the most valuable samples for labeling to reduce labeling costs. We designed a practical active learning method that adaptively allocates labeling resources to the most valuable unlabeled samples and the most likely mislabeled labeled samples, thus significantly reducing the overall labeling cost. We prove that the probability of our proposed method labeling more than one sample from any redundant sample set in the same batch is less than 1/k, where k is the number of the k-fold experiment used in the method, thus significantly reducing the labeling resources wasted on redundant samples. Our proposed method achieves the best level of results on benchmark datasets, and it performs well in an industrial application of automatic optical inspection.

## Introduction

In recent years, deep neural networks have been widely used. To train a deep neural network well, a large number of labeled samples are usually required. Commonly used benchmark datasets, such as MNIST (LeCun et al., 1998), CIFAR (Krizhevsky, 2009), and ImageNet (Krizhevsky, Sutskever & Hinton, 2012), contain tens of thousands or even millions of labeled samples. These labels are usually provided by human annotators and require high labor costs. For datasets in professional domains such as Automated Optical Inspection (AOI) (Abd Al Rahman & Mousavi, 2020), the labeling task usually needs to be performed by domain experts, and the scarcity and busyness of domain experts make the labeling task costly. For datasets in non-professional domains, crowdsourcing platforms, such as Amazon Mechanical Turk, are often used to perform labeling tasks. In crowdsourced labeling, it is difficult to guarantee the quality of the label, so additional inspections are required to control the quality of the label, which significantly increases the labeling cost. For example, in order for the final labeling accuracy to meet the desired requirements, each sample in the ImageNet dataset requires 2–5 annotators to label independently (Li, 2010), which increases the sample labeling cost by 1–4 times.

To reduce the cost of labeling samples, active learning has been widely studied. Active learning allows an algorithm to choose which samples to label, whereas supervised learning requires labels for all samples. Active learning aims to use as few labeled samples as possible to achieve high accuracy, thereby minimizing the cost of obtaining labeled samples. As the number of labeled samples increases, the performance of active learning will eventually converge to that of supervised learning. Common active learning methods can be divided into stream-based methods and pool-based methods. When a new sample is received, stream-based methods must decide whether to label the sample or ignore it. It is typically used in situations where unlabeled samples can be acquired continuously, such as cameras in self-driving cars that can take pictures continuously. Pool-based active learning methods typically start with a small set of labeled samples and a large set of unlabeled samples (Kumar & Gupta, 2020). Then, these methods iteratively train a model with labeled samples and use the model to select unlabeled samples for labeling until the model accuracy meets the requirements or reaches the budgeted limit for labeling cost. To increase speed, a batch of unlabeled samples is typically selected at each iteration, so the similarity between samples within the batch needs to be reduced (Kirsch, Van Amersfoort & Gal, 2019). In this article, we study pool-based methods for classification problems. Common pool-based active learning methods for classification problems include uncertainty (Settles, 2009), variation ratio (Beluch et al., 2018), and core-set (Sener & Savarese, 2018).

Noisy labeling results are often obtained due to annotator oversight or difficulty in distinguishing samples. For example, labeling errors exist in datasets such as MNIST and ImageNet (Northcutt, Jiang & Chuang, 2021). Most active learning does not care about label quality control, and it is assumed that all labels have passed quality control, but this leads to a waste of labeling resources. When the labeled sample size is small, a large number of low-quality labeled samples can lead to better performance than a small number of high-quality labeled samples; when the labeled sample size is large, improving the quality of existing labels can help to further improve performance. Combining active learning with label quality control can help reduce the cost of labeling. Active learning methods such as bidirectional active learning (BDAL) (Zhang, Wang & Yun, 2015) also take into account the presence of noise in the labels, but they require fast computation of model parameters after sample labels are changed, making it difficult to apply to methods with long training times, such as deep neural networks. Other methods, such as active learning from imperfect labelers (ALIL) (Yan, Chaudhuri & Javidi, 2016), require exponential computation as the input dimensionality increases, making it difficult to apply to high-dimensional samples such as images.

We combine active learning with label quality control to reduce the overall cost by adaptively allocating labeling resources for both active learning and quality control in each iteration. If we treat the annotator as a “model”, for quality control purposes we select samples that are prone to be mispredicted by this “model” and then check their labels. This is similar to the goal of active learning: select samples that are prone to misprediction and label them. We devise a metric to measure whether a sample is prone to misprediction or mislabeling. Based on the same metric, we can adaptively allocate labeling resources for active learning and label quality control. We design a practical algorithm based on this metric and prove that the algorithm reduces the cost of redundant labeling by labeling multiple samples from any redundant sample set with less than 1/*k* probability in one iteration, where k is the number of fold experiments that can be arbitrarily chosen in the method. By adaptively adjusting the allocation ratio of labeling resources as the number of labeled samples increases, the algorithm significantly improves the efficiency of labeling resource utilization, thus reducing the overall labeling cost. Our proposed method not only outperforms the compared methods on the benchmark dataset, but also performs well in real industrial applications.

The main contributions of this work are threefold:

- We propose a method that can adaptively allocate labeling resources for active learning and label quality control, and design a practical algorithm for it.
- We prove that the algorithm only labels multiple similar samples in the same batch with small probability, thus saving labeling resources.
- Our proposed method achieves state-of-the-art results on benchmark datasets and performs well in a real industrial application.

## Related work

In this section, we first introduce the pool-based active learning methods, then introduce the active learning methods with noisy labels, and finally introduce the label quality control methods.

### Pool-based active learning

**Uncertainty:** Let the classification probability vector predicted by model *M* for sample $x$ be $P = {({p_1}, \ldots ,{p_q})^T}$. Then, the uncertainty method labels samples with high entropy $H(P)$.

(1) $$H(P) = - \sum\limits_{i = 1}^q {{p_i}} \log {p_i}.$$

When the number of categories $q$ is 2, the uncertainty method degenerates to select the sample with ${p_1}$ closest to 0.5, which is equivalent to the active learning metric used in the dataset alignment active learning (Ben et al., 2022) method.

**CEAL:** The cost effective active learning (CEAL) (Wang et al., 2016) method improves the uncertainty method. It not only labels samples with high entropy values but also pseudolabels samples whose entropy values are less than a given threshold. CEAL methods benefit from the use of unlabeled samples, but choosing an appropriate threshold is difficult.

**Variation ratio:** The variation ratio method trains $c$ neural networks with different random initializations. They have the same network structure and use the same training set. The variance ratio method labels samples with large differences $V(x)$ between the predictions of $c$ neural networks.

(2) $$V(x) = 1 - {{{f_m}(x)} \over c},$$

where ${f_m}(x)$ is the number of predictions falling into the modal class for sample $x$. The variation ratio method performs well but requires additional computing resources to train multiple neural networks.

**Core-set:** The core-set method selects the labeled samples such that the spheres centered on the samples in the labeled dataset cover all samples. Let the labeled sample set be *L* and the unlabeled sample set be *U*. *T* is the sample set that needs to be labeled, and $\lambda \,({x_1},{x_2})$ is the distance between samples ${x_1}$ and ${x_2}$. The core-set method chooses *T* to minimize

(3) $${\max _{{x_i} \in U,{x_j} \in L \cup T}}\lambda ({x_i},{x_j}).$$

The core-set method has an intuitive geometric interpretation, but it is often difficult to choose a suitable definition of the distance $\lambda\, ({x_i},{x_j})$.

**BatchBALD:** The BatchBDAL (Kirsch, Van Amersfoort & Gal, 2019) method selects a set of samples to minimize the uncertainty of the model parameters. With appropriate transformations, it chooses to label a set of samples ${x_{1:b}}$ to maximize

(4) $${\cal H}({y_{1:b}}|{x_{1:b}},{D_{train}}) - {E_{p(w|{D_{train}})}}{\cal H}({y_{1:b}}|{x_{1:b}},w,{D_{train}}),$$

where ${\cal H}$ denotes the conditional entropy. The BatchBDAL method is the original BDAL (Gal, Islam & Ghahramani, 2017) method when $b$ is 1. The BatchBDAL method reduces the probability of labeling multiple similar samples in the same batch, which results in better performance.

**Learning loss:** The learning loss (Yoo & Kweon, 2019) method adds a loss prediction model to the task model. The model takes the features from the task model as input and then predicts the loss value of the task model for each sample. The learning loss method is widely applicable to various tasks, but its loss prediction model is closely related to the structure of the task model, so the loss prediction model needs to be redesigned when using different task models, which increases the workload of using it.

**VAAL:** The Variational Adversarial Active Learning (VAAL) (Sinha, Ebrahimi & Darrell, 2019) method first trains an auto-encoder using labeled and unlabeled samples, and then trains a discriminator to predict whether a sample is already labeled based on the features extracted by the auto-encoder. If an unlabeled sample is predicted to be labeled by the discriminator, the variational adversarial method believes that the sample can be well represented by the existing labeled samples and therefore does not deserve to be labeled. The variational adversarial method chooses to label those unlabeled samples that the discriminator is confident are unlabeled.

**EADA:** The Energy-based Active Domain Adaptation (EADA) (Xie et al., 2022) method is concerned with the application of active learning to knowledge transfer in the target domain. It combines domain features and instance uncertainty to select samples to be labeled. The EADA method also reduces the domain gap by compactly aligning the free energy of the target domain around the source domain through regularization terms. The EADA method reduces the labeling cost required to solve the domain adaptation problem and also expands the application scenario of active learning.

More active learning methods can be found in these surveys (Kumar & Gupta, 2020; Ren et al., 2021).

### Active learning with noisy labels

**BDAL:** The BDAL method calls the active learning process of selecting samples for labeling the forward process, and adds the backward process of selecting samples for inspection. In the backward process, the BDAL method checks whether the label of sample $x_{BL}^*$ is correct, where

(5) $$x_{BL}^* = \mathop {\arg \min }\limits_{x \in L} \sum\limits_i P ({y_i}|x;{\theta _{L \setminus (x,y_i^*)}}) \cdot \sum\limits_{{x^u} \in U - x} H ({y^u}|{x^u};{\theta _{L \setminus (x,y_i^*)}}).$$

${\theta _{L \setminus (x,Y_i^*)}}$ represents the model parameters trained after removing sample $x$ and its label $y_i^*$ in the labeled sample set *L*. The BDAL method combines active learning and label quality control but requires multiple recalculations of model parameters after the training set changes, which is difficult to apply to deep neural networks.

**ALIL:** The ALIL method allows annotators to not label indeterminate samples and has satisfactory query complexity in specific cases. However, its computations grow exponentially as the sample dimension increases, which is difficult to apply to sample types such as images.

### Label quality control

**Simple method:** The simple label quality control method gives each sample to multiple annotators for independent labeling, and then takes the most frequent of the multiple label results as the final label result. Although this method increases the labeling cost several times, it is widely used (Li, 2010) because it is easy to implement and is independent of the chosen machine learning method.

**Confident learning:** The confident learning (Northcutt, Jiang & Chuang, 2021) method first estimates the joint distribution matrix of the noise label and the true label based on the predicted probability output by the model, and then selects the samples that may be mislabeled based on the distribution matrix. For example, the distribution matrix indicates that a sample of class A is mislabeled as B with probability $p$; then, for the sample labeled as B, the confident learning method considers the first $n*p$ samples most likely to belong to class A to be mislabeled, where $n$ is the sample size.

**GLAD:** The Generative model of Labels, Abilities, and Difficulties (GLAD) (Whitehill et al., 2009) method better aggregates the label results of multiple independent annotators. Different from the traditional method that uses the label supported by the most annotators as the final label, the GLAD method establishes a probability model to analyze the difficulty of labeling the sample and the professional level of the annotator, thereby obtaining more accurate label results.

## Proposed approach

In this section, we first combine active learning with label quality control to adaptively allocate labeling resources. Then, we propose a practical algorithm for our approach. Finally, we prove that the algorithm is less affected by redundant samples.

### Active learning with label quality control

Both active learning and label quality control consume labeling resources. If all labeling resources are allocated to active learning, the performance of the model may be limited by samples with low label quality. If each sample is labeled multiple times to fully inspect its label quality, the same labeling resources will yield fewer high-quality labeled samples, which will also limit the performance of the model. When the number of labeled samples is small, we tend to increase the number of labeled samples; when the number of labeled samples is sufficient, we tend to improve the label quality of the labeled samples. Therefore, allocating labeling resources to active learning and label quality control in a fixed proportion will not achieve the best performance, and we need to allocate labeling resources adaptively with the training progress.

Active learning and label quality control typically use different metrics for sample selection. Therefore, it is difficult to evaluate whether the benefit of labeling a new sample is greater than the benefit of inspecting an existing label. If active learning and label quality control use the same sample selection metric, we can adaptively allocate labeling resources based on this metric. A human annotator can be considered as a model whose predictions may be wrong due to oversight or other reasons. The goal of label quality control is to inspect samples that the model (human annotator) may predict incorrectly, which is similar to the goal of active learning. Based on this, we propose a sample selection metric that can be used for both active learning and label quality control.

If we let the label space be ${\cal Y}$, within this space, the actual label of sample $x$ is $y$, the label given by the annotator is $\bar y$, and the prediction given by model *M* is $\hat y$. Let $D\,({y_1},{y_2})$ be the distance in the label space. The smaller the distance is, the closer ${y_1}$ is to ${y_2}$. We call ${d_{pre}} = D\,(y,\hat y)$ the predicted distance, ${d_{ann}} = D\,(y,\bar y)$ is the annotation distance, and ${d_{obs}} = D\,(\bar y,\hat y)$ is called the observation distance. For active learning, we choose to label samples with a large predicted distance ${d_{pre}}$; to control the label quality, we choose to inspect samples with a large annotation distance ${d_{ann}}$. Therefore, to combine active learning and label quality control, we choose to label samples with a large $\max \,({d_{pre}},{d_{ann}})$.

**Figure 1:**
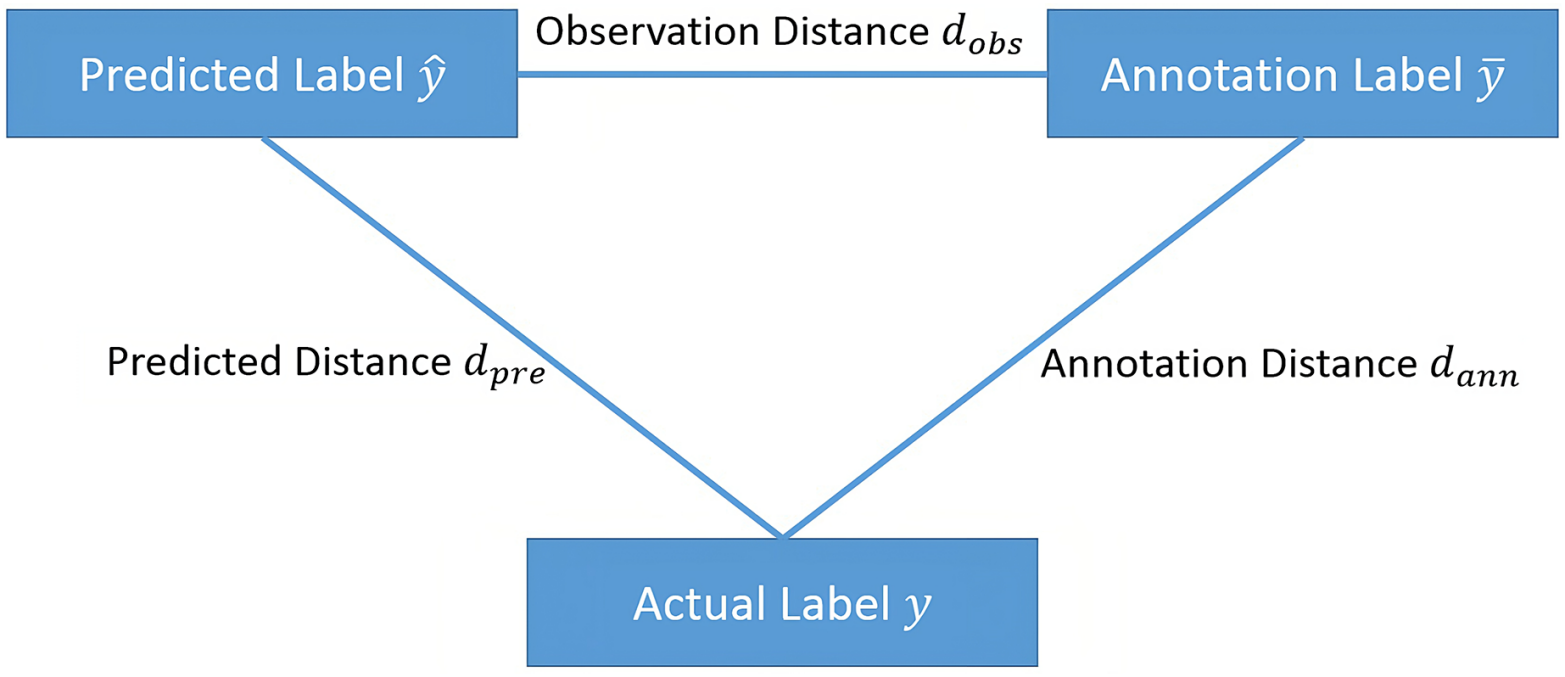
The relationships between observation distance, annotation distance and predicted distance.

Since the actual label $y$ of the sample $x$ is usually unknown, it is difficult to obtain the value of $\max \,({d_{pre}},{d_{ann}})$. If the distance $D\,({y_1},{y_2})$ in the sample space ${\cal Y}$ satisfies the triangle inequality, as shown in Fig. 1, then

(6) $$\max ({d_{pre}},{d_{ann}}) \ge {1 \over 2}{d_{obs}}.$$

Therefore, we choose to label or inspect samples with a large observation distance ${d_{obs}}$, which represents the lower bound of the predicted distance ${d_{pre}}$ and the annotation distance ${d_{ann}}$.

For various common machine learning tasks, such as classification and regression, the label space ${\cal Y}$ usually belongs to a high-dimensional vector space. The ${L_1}$ distance and ${L_2}$ distance, *etc*. in the high-dimensional vector space satisfy the triangular inequality, and we usually choose the ${L_1}$ distance for the stability of the gradient in the training process. For example, in the q-classification problem, the elements in the label space ${\cal Y} = \{ {({p_1}, \ldots ,{p_q})^T}:0 \le {p_i} \le 1,1 \le i \le q;\sum\nolimits_{i = 1}^q {{p_i}} = 1\}$ are all possible classification probability vectors. Since the sample is difficult to distinguish, the actual label $y$ is not necessarily a one-hot vector, but the label $\bar y$ given by the annotator is often a one-hot vector. We use the ${L_1}$ distance as the distance metric *D* in the label space ${\cal Y}$, and then define the observation distance ${d_{obs}}$, annotation distance ${d_{ann}}$, and prediction distance ${d_{pre}}$.

### Practical algorithm

To apply our proposed method to practical problems, two issues need to be addressed. First, each sample must have a label. Second, neural networks often overfit the training set and thus underestimate the observation distance.

To make each sample have a label, we introduce the pseudolabel method. After updating the labeled sample set, we first train the model on the labeled sample set, and then use the trained model to assign a pseudolabel to each sample in the unlabeled sample set. Pseudolabel methods are widely used in semisupervised learning and are widely applicable to various problems.

To obtain a more accurate observation distance, the model *M* used to calculate the observation distance of the unlabeled sample $x$ should be trained on a dataset that does not contain $x$. To reduce the number of calculations, we randomly divide the entire dataset (including the labeled dataset and the unlabeled dataset with pseudolabels) into $k$ groups. Each time, training is performed on $k - 1$ of the groups, and the trained model is used to calculate the observation distance on the remaining group.

**Figure 2:**
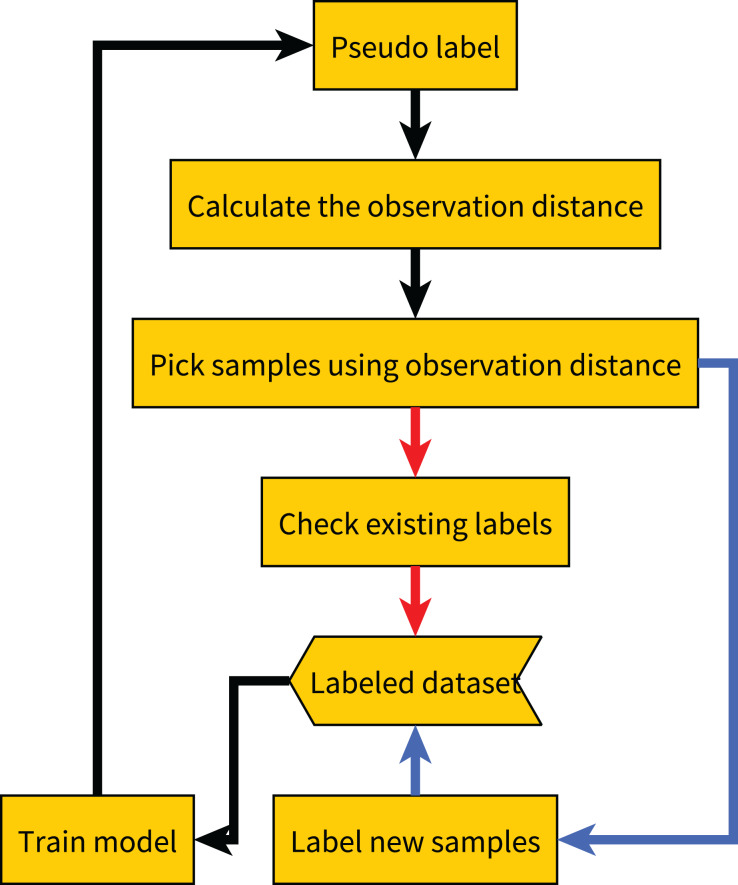
Flow chart of our proposed method. The red line indicates the label quality control process, and the blue line indicates the active learning process.

After obtaining the observation distances of all samples, we select the sample with the largest observation distances. If the sample has already been labeled, we re-label it and then update the label based on the results of all previous labels. For example, we update the label to the most frequent label among all previous results. If the sample has not been labeled, we label it and add it to the labeled dataset. The complete process is shown in Algorithm 1, and the flowchart of the proposed method is shown in Fig. 2.

**Algorithm 1:** Active learning with label quality control.

**Input: **Unlabeled samples *U*, initially labeled samples *L*, batch size *b*, maximum iteration number *N*, number of folds *k*
**Output: **Model *M*
**for** $i \leftarrow 1$ **to** *N* **do**
Train model ${M^L}$ on *L*;
Use model ${M^L}$ to pseudolabel the sample in *U* to get the pseudolabeled samples ${L^U}$;
Let $\hat L = L \cup {L^U}$, then randomly divide $\hat L$ into *k* subset $\{ {\hat L_1}, \ldots ,{\hat L_k}\}$ of the same size;
**foreach** ${\hat L_j}$ **do**
Train model *M*_*j*_ on $\hat L/{\hat L_j}$;
Use model *M*_*j*_ to calculate the observation distance $\tilde d$ of the sample in ${\hat L_j}$;
**end**
Choose *b* samples from $\hat L$ with the largest $\tilde d$ as the samples *T*;
**foreach ** $x \in T$ **do**
**if** $x \in L$ **then**
Label *x* again, then update the label of *x*;
**end**
**if** $x \in U$ **then**
Label *x*, add *x* to *L*, and remove *x* from *U*;
**end**
**end**
**end**
*M* = Ensemble ( ${M_1}, \ldots ,{M_k}$);
**return** *M*

### Algorithmic property

Next, we prove that our proposed algorithm is less affected by redundant samples in the dataset.

**Theorem 1.** *If there are duplicate samples in the unlabeled dataset $U = \{ {x_1}, \ldots ,{x_n}\}$, we refer to ${U_R} = \{ {x_{R(1)}}, \ldots ,{x_{R(m)}}\} \subseteq U$ as a repetitive subset of U, where ${x_{R(1)}} = {x_{R(2)}} = \cdots = {x_{R(m)}}$, $m \ge 2$. If the neural network has sufficient fitting ability, for any ${U_R}$, the sample set T to be labeled selected by Algorithm 1 satisfies $P(|T \cap {U_R}| \ge 2) \le 1/k$, where $k$ is the number of folds when calculating the observation distance*.

*Proof*. In the folding experiment, if the samples in ${U_R}$ are divided into different groups, the samples in ${U_R}$ will appear in both the training set and the sample set used to calculate the observation distance. Because the neural network is fully fitted, the observation distance of the sample is 0, so it will not be selected for labeling. In summary, it may be selected for labeling only when the samples in ${U_R}$ are divided into the same group, hence

(7) $$P(|T \cap {U_R}| \ge 2) \le {1 \over {{k^{m - 1}}}} \le {1 \over k}.$$

If the number of folds $k$ is 5, then for any redundant sample group ${U_R}$, the probability that there are redundant samples from ${U_R}$ in the sample set to be labeled by Algorithm 1 does not exceed $1/5$. It can be seen that our method avoids labeling redundant samples. Although the proof is only for redundant samples, the experiment in Section “Experiments of Active Learning” shows that our proposed method also performs well for similar samples.

## Experimental results

First, we conduct experiments under noisy labels to evaluate the performance of our proposed method for adaptive allocation of labeling resources. Then, we compare with several active learning methods and verify the anti-redundancy of our proposed method to evaluate the active learning performance of our proposed method. Finally, we compare our proposed method with the mainstream label quality control methods to evaluate the label quality control performance of our proposed method.

### Experiments under noisy label

We first describe the experimental settings used and then compare it to the no quality control, full quality control, and fixed ratio quality control methods.

#### Experimental settings

We conduct experiments on the MNIST dataset based on simulated labeling noise. Due to the difficulty of obtaining the original noisy label results, we use the labels provided in the MNIST dataset as the actual labels and simulate the labeling noise on the MNIST dataset. We assume that the annotator has a 95% probability of providing the actual label and a 5% probability of providing an incorrect label. For example, if the actual label is the number 0, the probability that the label result is the number 0 is 95%, and the probability that the label result is any other number is 1/180.

All images are resized to 32 × 32 and no data augmentation is used. We use the LeNet5 (LeCun et al., 1998) network and the cross-entropy loss function. We use SGD optimizer and set the batch size to 32, then train 1,000 epochs. The initial learning rate is set to 0.1, momentum is set to 0.9, weight decay is set to 1e−4, and the learning rate is adjusted to 0.01 after 500 epochs. To prevent overconfidence in the classification probability vector output by the neural network, we use temperature scaling (Hinton, Vinyals & Dean, 2015) method to improve the performance of the model after the model training is completed. In the k-fold experiment, we use the set of data not used for training to calculate the optimal temperature.

We randomly select 100 samples from the training set of the MNIST dataset as the initial set of labeled samples, and in each iteration, we label or inspect 100 samples until we have consumed 1,000 labeling resources. We perform 10 random experiments, each with different initial 100 random samples, and then take the average of the results of the 10 experiments as the final result. The methods used for comparison include: “ALQC” is our proposed method, “CEAL” is the cost effective active learning method, “Core” is the core-set method, “Rand” is the method of randomly selecting samples to be labeled, “Var” is the variation ratio method, and “Unc” is the uncertainty method. See Section “Related Work” for details on these methods.

#### Compare with no quality control

**Figure 3:**
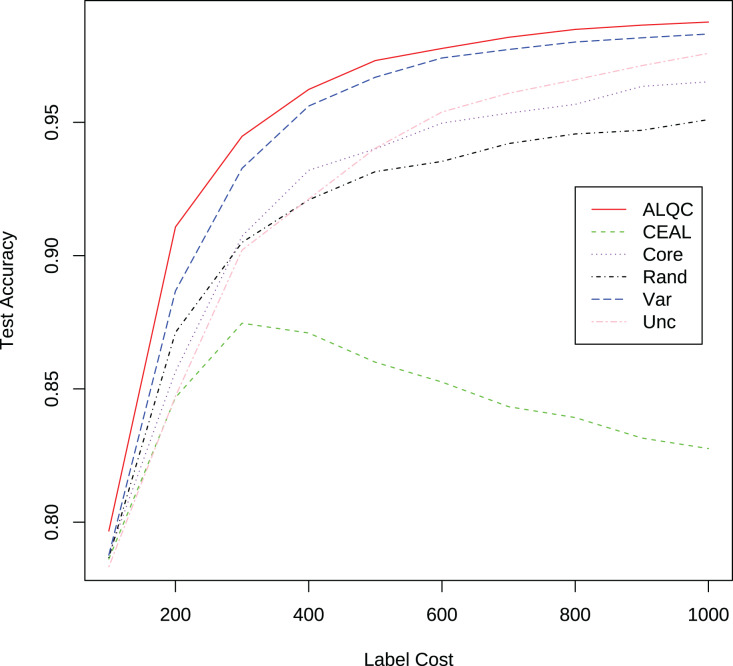
Experimental results of active learning methods without label quality control on the MNIST dataset.

We first compare with active learning methods without label quality control. These active learning methods directly use low-quality labeled datasets thus obtain more labeled samples. The experimental results in Fig. 3 and Table 1 show that our proposed ALQC method outperforms the other methods. When consuming the same label cost, the ALQC method achieves higher test set accuracy than other methods, thus achieving optimal efficiency in the use of labeling resources. The excellent performance of the ALQC method shows that ignoring label quality control and directly using low-quality labels does not achieve the best performance. This highlights the necessity of combining active learning with label quality control. The CEAL method is limited by the large number of false pseudolabels and therefore performs poorly, which also illustrates the importance of label quality control in active learning.

**Table 1:** Test set accuracy of active learning methods without label quality control on the MNIST dataset.

Label cost	ALQC	CEAL	Core	Rand	Var	Unc
100	0.7967	0.7862	0.7877	0.7868	0.7877	0.7833
200	0.9107	0.8468	0.8565	0.8712	0.8867	0.8472
300	0.9447	0.8747	0.9073	0.9050	0.9328	0.9021
400	0.9623	0.8710	0.9320	0.9209	0.9561	0.9212
500	0.9732	0.8600	0.9400	0.9315	0.9669	0.9403
600	0.9777	0.8526	0.9497	0.9353	0.9742	0.9539
700	0.9819	0.8433	0.9535	0.9420	0.9773	0.9609
800	0.9849	0.8393	0.9567	0.9456	0.9801	0.9660
900	0.9865	0.8316	0.9635	0.9470	0.9817	0.9713
1,000	0.9876	0.8276	0.9652	0.9510	0.9831	0.9759

#### Compare with fully quality control

**Figure 4:**
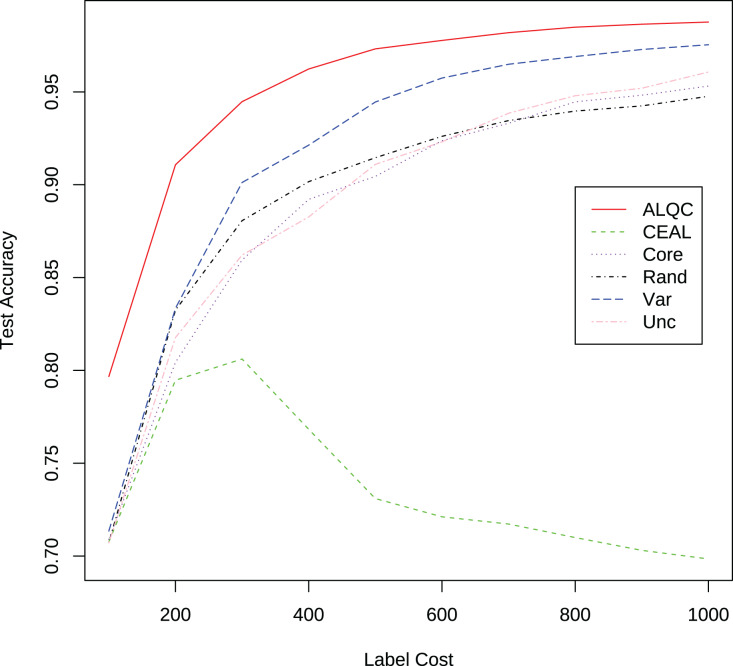
Experimental results of active learning methods with the simple label quality control method on the MNIST dataset.

We then compare our proposed method with active learning methods that use a simple label quality control method for label quality control. The simple label quality control method requests multiple labels for each sample until a category appears twice in the label results. Then we select that category as the final label result. If the method does not stop after five times of labeling, one of the five labeling results is randomly selected as the final labeling result. All samples are first run through this simple method to control the labeling quality and then used for active learning, so the average number of annotations required to obtain a labeled sample is 2.1. The accuracy of the labeling results obtained from this process is greater than 99.9%. The results in Fig. 4 and Table 2 show that our proposed method also outperforms other methods. The ALQC method uses fewer labeling resources than other methods to achieve the same test set accuracy, thus reducing labeling costs. The excellent performance of the ALQC method shows that forcing all samples to be labeled with high quality does not achieve the best performance either. Therefore, it is necessary to properly allocate labeling resources between active learning and label quality control.

**Table 2:** Test set accuracy of active learning methods with the simple label quality control method on the MNIST dataset.

Label cost	ALQC	CEAL	Core	Rand	Var	Unc
100	0.7967	0.7076	0.7090	0.7073	0.7135	0.7071
200	0.9107	0.7947	0.8043	0.8325	0.8335	0.8177
300	0.9447	0.8061	0.8595	0.8806	0.9012	0.8621
400	0.9623	0.7683	0.8921	0.9017	0.9213	0.8826
500	0.9732	0.7310	0.9045	0.9145	0.9445	0.9110
600	0.9777	0.7211	0.9237	0.9261	0.9575	0.9231
700	0.9819	0.7173	0.9331	0.9345	0.9649	0.9385
800	0.9849	0.7100	0.9447	0.9397	0.9690	0.9479
900	0.9865	0.7031	0.9482	0.9425	0.9728	0.9520
1,000	0.9876	0.6985	0.9531	0.9476	0.9754	0.9607

#### Compare with fixed ratio quality control

**Figure 5:**
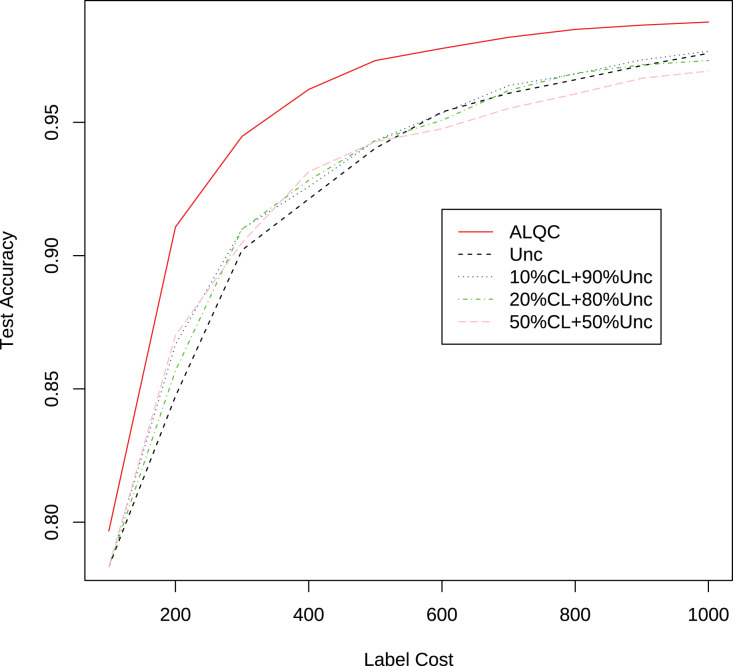
Experimental results of the uncertainty method combined with the confident learning method on the MNIST dataset.

**Figure 6:**
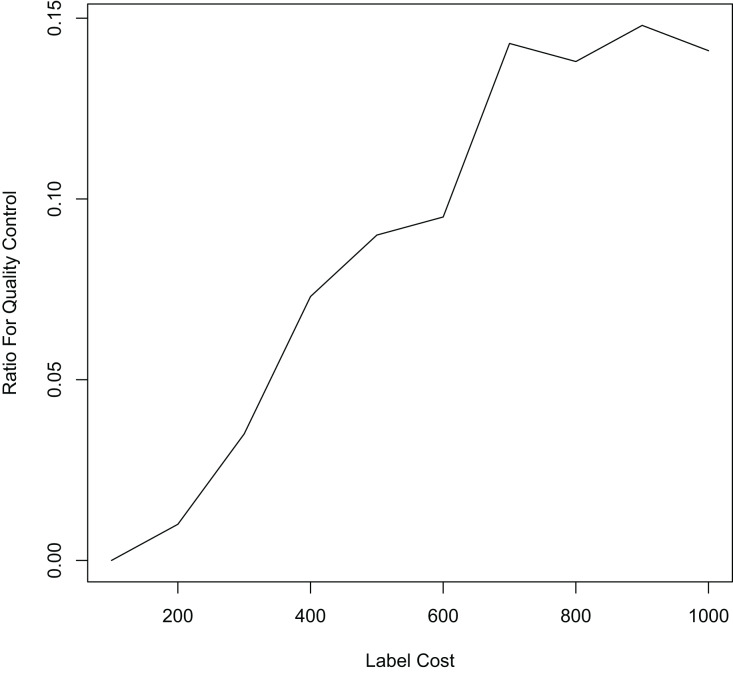
The ratio of labeling resources allocated to label quality control by the ALQC method on the MNIST dataset.

Finally, we compare the proposed method with methods that allocate labeling resources to active learning and label quality control in a fixed ratio. In each iteration, we allocate 10%, 20%, and 50% of the labeling resources to the confident learning method to improve label quality, and the remaining labeling resources to the uncertain method to select samples worth labeling. The experimental results in Fig. 5 and Table 3 show that our proposed method still outperforms the other methods. This shows that simply allocating a fixed ratio of labeling resources to improve labeling quality does not achieve the best performance, and highlights the importance of adaptively allocating labeling resources. Our proposed ALQC method is based on the same sample selection metric, and thus can adaptively allocate labeling resources to achieve the best performance. As can be seen in Fig. 6, the ALQC method allocates almost 0% of the labeling resources to inspect the quality of existing labels at the beginning, and adaptively increases the allocation percentage to about 15% as the labeling sample size increases, demonstrating a good adaptive labeling resource allocation capability.

**Table 3:** Test set accuracy of the uncertainty method combined with the confident learning method on the MNIST dataset.

Label cost	ALQC	Unc	10% CL + 90% Unc	20% CL + 80% Unc	50% CL + 50% Unc
100	0.7967	0.7833	0.8008	0.7916	0.8030
200	0.9107	0.8472	0.8667	0.8569	0.8700
300	0.9447	0.9021	0.9100	0.9099	0.9049
400	0.9623	0.9212	0.9258	0.9282	0.9314
500	0.9732	0.9403	0.9431	0.9432	0.9427
600	0.9777	0.9539	0.9535	0.9507	0.9476
700	0.9819	0.9609	0.9638	0.9619	0.9552
800	0.9849	0.9660	0.9681	0.9684	0.9606
900	0.9865	0.9713	0.9734	0.9716	0.9665
1,000	0.9876	0.9759	0.9767	0.9732	0.9692

The above experiments show that adaptive allocation of labeling resources can achieve better performance compared to no quality control, full quality control, and fixed ratio quality control. By allocating the labeling resources to the highest value samples to be labeled and samples to be inspected, our proposed ALQC method improves the utilization of labeling resources and thus achieves the best performance.

### Experiments of active learning

Assuming that the labeling is absolutely correct, we compare the proposed method with various active learning methods on the MNIST dataset and the CIFAR-10 dataset to observe its active learning performance, and experimentally observe the redundancy resistance of our proposed method.

**Figure 7:**
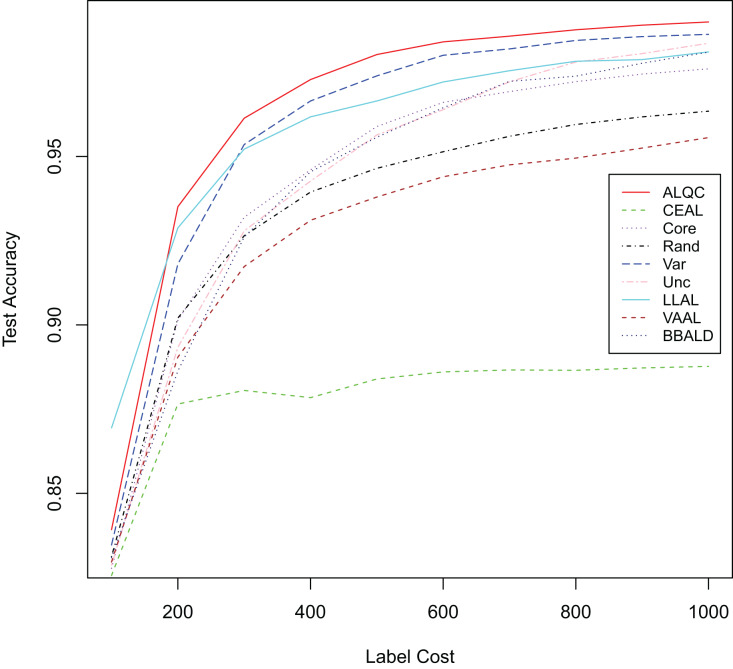
Experimental results without label noise on the MNIST dataset.

First, we experiment with the MNIST dataset. The experimental settings are the same as in the “Experimental Settings” section. Instead of simulating the noisy noise, we use the labels provided by the dataset directly. We add comparisons with the recent active learning methods: “LLAL” is the learning loss method, “VVAL” is the variational adversarial active learning method, and “BBALD” is the BatchBALD method. We use the code provided by the authors of the relevant articles for our experiments and try to make the training settings as similar as possible. The network structure, the data augmentation methods, the way and number of initial sample sets are selected, the number of labeled samples per iteration, and the forbidding of additional labeled validation sets are all consistent with our experimental settings. The experimental results are shown in Fig. 7 and Table 4. It can be seen that our proposed ALQC method outperforms other methods on the active learning task without noise labeling.

**Table 4:** Test set accuracy of active learning methods without label noise on the MNIST dataset.

Label cost	ALQC	CEAL	Core	Rand	Var	Unc	LLAL	VAAL	BBALD
100	0.8392	0.8256	0.8278	0.8313	0.8347	0.8287	0.8694	0.8296	0.8309
200	0.9351	0.8765	0.9013	0.9020	0.9181	0.8934	0.9288	0.8904	0.8863
300	0.9614	0.8805	0.9319	0.9265	0.9536	0.9279	0.9522	0.9173	0.9261
400	0.9728	0.8784	0.9460	0.9394	0.9665	0.9427	0.9618	0.9311	0.9454
500	0.9803	0.8840	0.9589	0.9465	0.9739	0.9564	0.9665	0.9380	0.9557
600	0.9840	0.8861	0.9661	0.9514	0.9800	0.9639	0.9721	0.9440	0.9645
700	0.9857	0.8866	0.9692	0.9560	0.9819	0.9722	0.9755	0.9475	0.9723
800	0.9876	0.8865	0.9722	0.9595	0.9845	0.9780	0.9783	0.9495	0.9738
900	0.9890	0.8872	0.9745	0.9618	0.9856	0.9805	0.9788	0.9525	0.9777
1,000	0.9899	0.8877	0.9760	0.9635	0.9863	0.9836	0.9810	0.9556	0.9810

**Figure 8:**
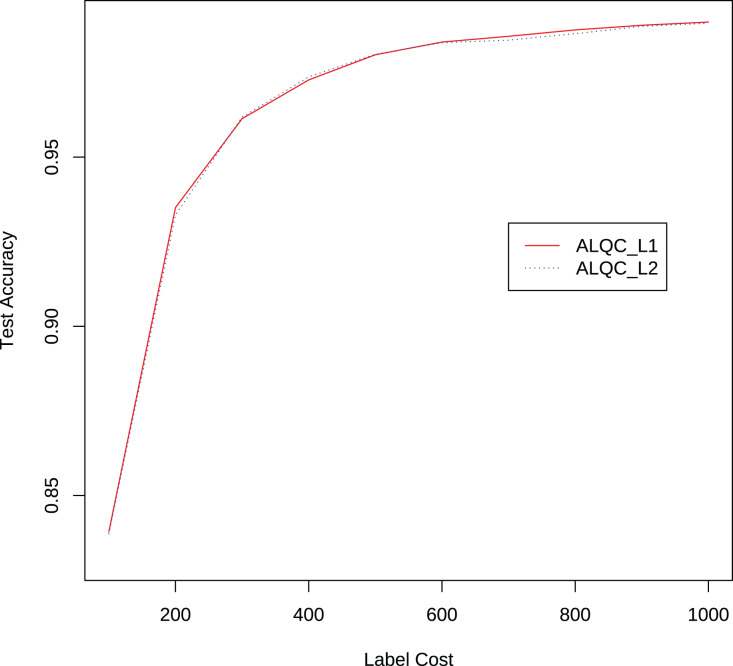
Experimental results of the ALQC method using ${L_1}$ and ${L_2}$ distance metrics in the MNIST dataset.

We also compare the performance of the ALQC method using the ${L_1}$ distance metric and the ${L_2}$ distance metric. The experimental results in Fig. 8 show that the ALQC method is robust to the choice of distance metric. The ALQC method relies only on the support of the distance metric for the triangular inequality and does not require a specially designed distance metric.

**Figure 9:**
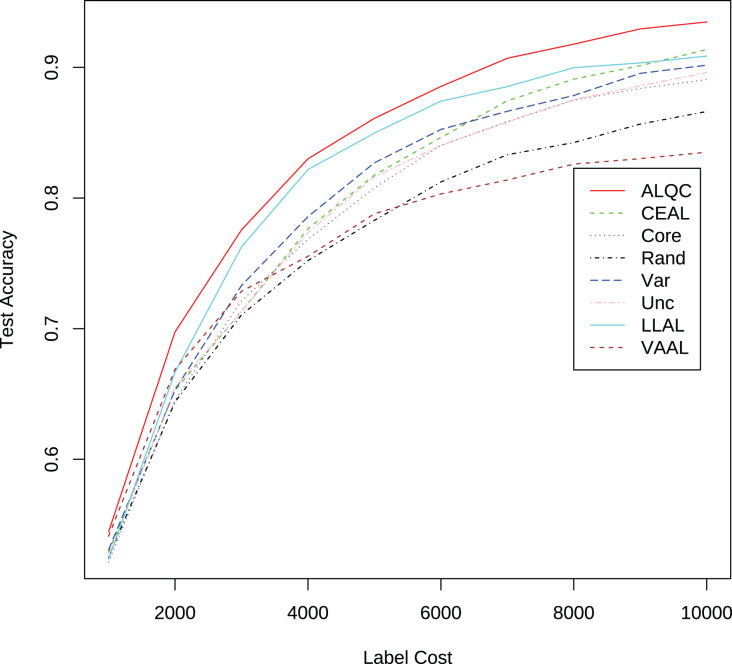
Experimental results without label noise on the CIFAR-10 dataset.

Then we conduct the experiments on the CIFAR-10 dataset. Most of the experimental settings are the same as in Section “Experimental Settings”, with the following main differences. We switch to use the ResNet-18 (He et al., 2016) network. We also use the SGD optimizer and update the batch size to 128, then train 200 epochs. The learning rate is adjusted to 0.2 times the previous one after 60, 120, and 160 epochs. We use RandomCrop, RandomHorizontalFlip, and Cutout (DeVries & Taylor, 2017) methods for data augmentation. We randomly select 1,000 samples from the training set in the CIFAR-10 dataset as the initial set of labeled samples, and for each iteration, we label or inspect 1,000 samples until we have consumed 10,000 labeling resources. The experimental results are shown in Fig. 9 and Table 5. It can be seen that our proposed method also outperforms the other methods.

**Table 5:** Test set accuracy of active learning methods without label noise on the CIFAR-10 dataset.

Label cost	ALQC	CEAL	Core	Rand	Var	Unc	LLAL	VAAL
1,000	0.5444	0.5289	0.5214	0.5250	0.5307	0.5261	52.3380	54.1160
2,000	0.6975	0.6528	0.6452	0.6440	0.6532	0.6553	66.6740	66.8880
3,000	0.7756	0.7133	0.7213	0.7106	0.7330	0.7132	76.2580	72.8600
4,000	0.8300	0.7765	0.7684	0.7521	0.7858	0.7738	82.1780	75.5400
5,000	0.8610	0.8176	0.8078	0.7829	0.8270	0.8163	84.9720	78.7960
6,000	0.8854	0.8463	0.8403	0.8123	0.8525	0.8403	87.4140	80.3160
7,000	0.9070	0.8744	0.8585	0.8332	0.8664	0.8582	88.5320	81.3820
8,000	0.9178	0.8910	0.8750	0.8425	0.8785	0.8752	89.9800	82.5960
9,000	0.9295	0.9013	0.8838	0.8566	0.8954	0.8860	90.3420	83.0160
10,000	0.9348	0.9136	0.8907	0.8662	0.9017	0.8960	90.8620	83.5040

**Figure 10:**
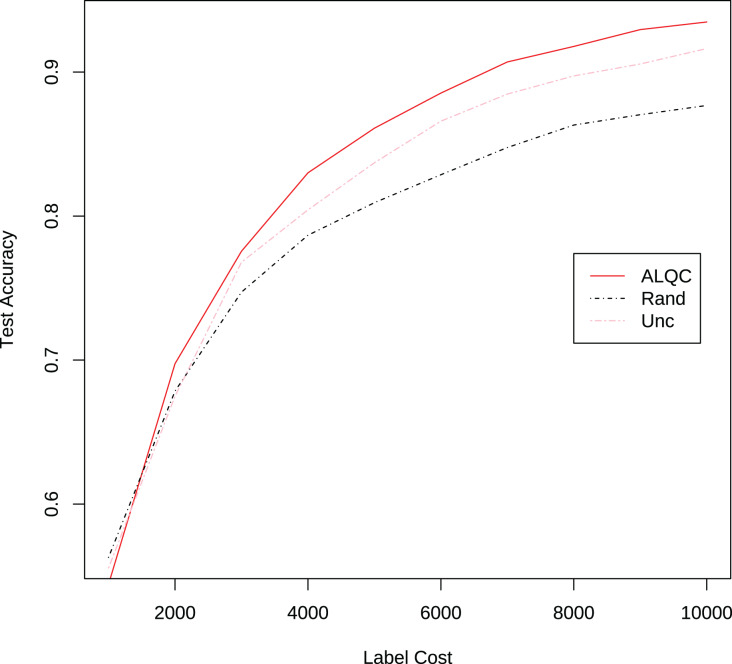
Experimental results on the CIFAR-10 dataset when using pseudolabeling.

Although our proposed method uses pseudolabels to calculate the observation distance, the performance improvement does not come from the use of pseudolabels. In the above experiments, our proposed method outperforms the CEAL method that also uses pseudolabels. This is because our proposed method keeps improving the labeling quality of the training set, but the CEAL method accumulates more and more incorrect pseudolabels, which limits its performance. We also add pseudolabels to the random and uncertain methods and compare them with our method. The experimental results are shown in Fig. 10. It can be seen that our proposed method still outperforms the other methods when using pseudolabels. Thus, the performance improvement mainly stems from the good redundancy resistance rather than simply using pseudolabels.

**Figure 11:**
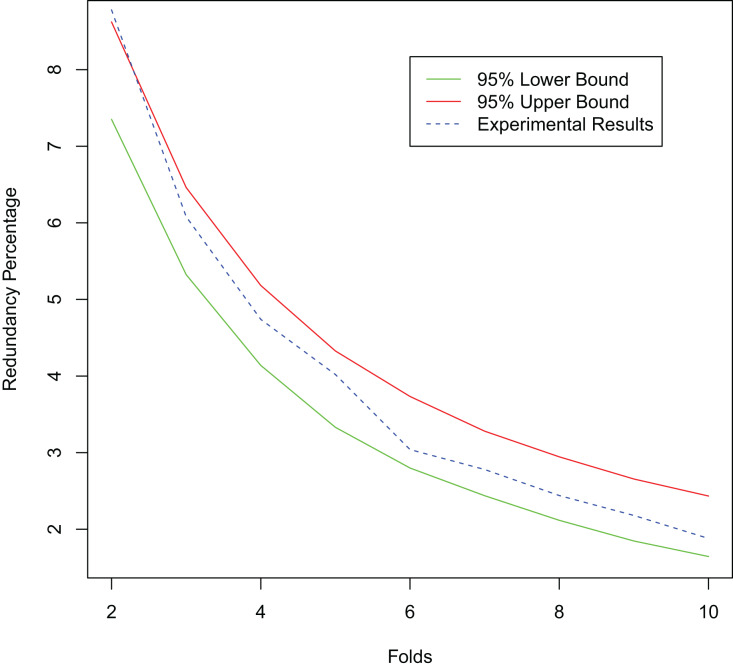
Experimental results for the redundant dataset.

Since our proposed method has excellent redundancy resistance, it reduces the labeling resources wasted on labeling multiple redundant samples and thus achieves better active learning performance. To further verify the redundancy resistance of our proposed method, we simulate the presence of redundant samples in the dataset. We randomly select 20% of the samples in the training set of the MNIST dataset to replicate, add random noise from a normal distribution N (0, 0.01) to the replicated samples, and then add them to the original dataset. Training starts with 100 randomly selected labeled samples and labels 100 new samples at each iteration until a total of 1,000 labeled samples are obtained. We test the redundant sample rate of the selected sample set under different numbers of folds $k$ and compare it with its 95% confidence interval. As shown in Fig. 11, with the increase of the number of folds, the redundancy rate of the selected sample set becomes lower. This is consistent with the conclusion in Theorem 1 that our method has good anti-redundancy performance.

The above experiments show that our proposed method has excellent active learning performance due to good redundancy resistance, and thus can achieve excellent performance in active learning tasks under noise labeling.

### Experiments of label quality control

**Figure 12:**
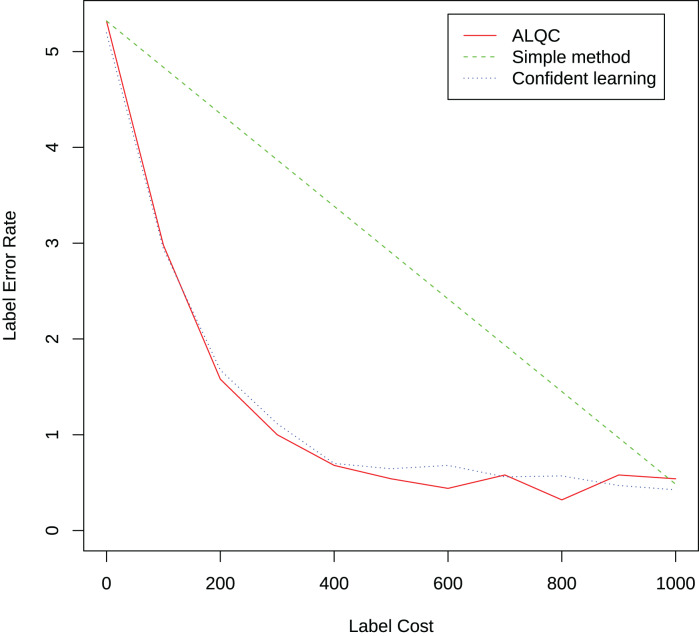
Experimental results of label quality control.

We randomly select 1,000 samples from the training set in the MNIST dataset and label them with 95% accuracy. We then use our proposed method to inspect samples that may be mislabeled to improve the quality of the labels. We inspect 100 existing labels per iteration until 1,000 inspections are performed. We compare it with the simple label quality method and the confidence learning method mentioned in the “Related Work” section. The result is shown in Fig. 12. It can be seen that our proposed method can select samples that may be mislabeled more effectively than the simple method and achieve similar performance as the confidence learning method.

The experimental results in this section show that our proposed method can allocate labeling resources to the most valuable tasks and has good active learning capability with labeling quality capability, thus achieving leading performance in active learning tasks under noisy labeling.

## Practical application

We introduce the proposed method into the Automated Optical Inspection (AOI) system, which solves the high labeling cost problem caused by extreme redundancy and data imbalance in the AOI system.

### Problem description

AOI is an optical-based detection system that uses images captured by a camera to detect defects in printed circuit boards (PCB). The AOI system is constantly receiving so many new unlabeled samples that there are not enough resources to label all of them. Therefore, it is necessary to use the active learning method to select samples worthy of being labeled.

The redundancy of the AOI dataset is extremely serious. There are multiple similar defect samples in the dataset. Theorem 1 shows that our proposed method can resist the interference of redundant samples, so we apply it to the AOI system.

### Method adaptation

**Figure 13:**
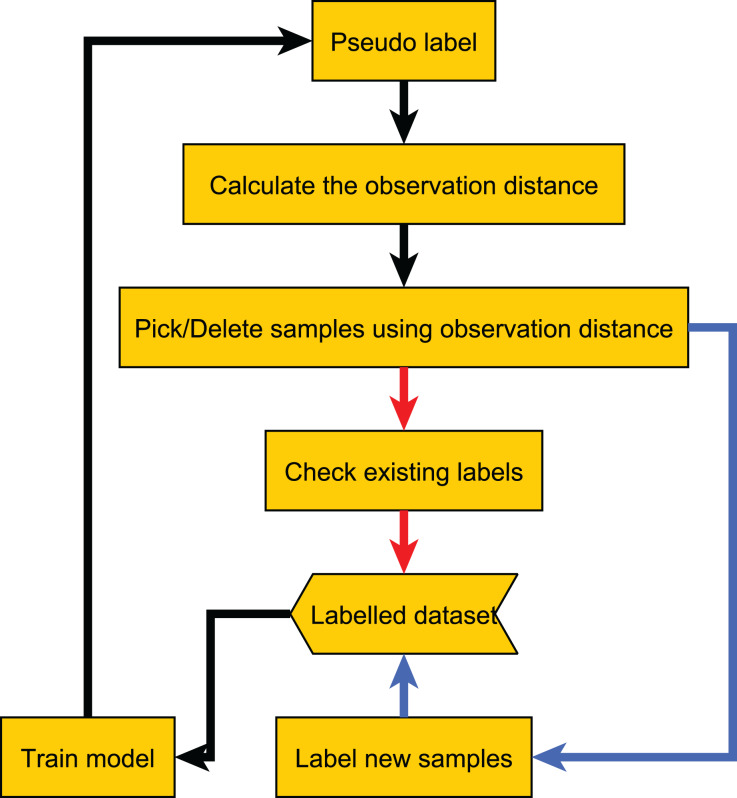
Flow chart of the adapted method.

Since too many redundant samples waste computational resources, we modify the original method workflow. Samples with a small observation distance mean that the current model can accurately classify them, so they are more likely to be redundant samples. After computing the observation distances, we permanently remove samples with sufficiently small observation distances from the unlabeled dataset, thereby speeding up the method. The adapted workflow is shown in Fig. 13.

### Results and analysis

**Figure 14:**
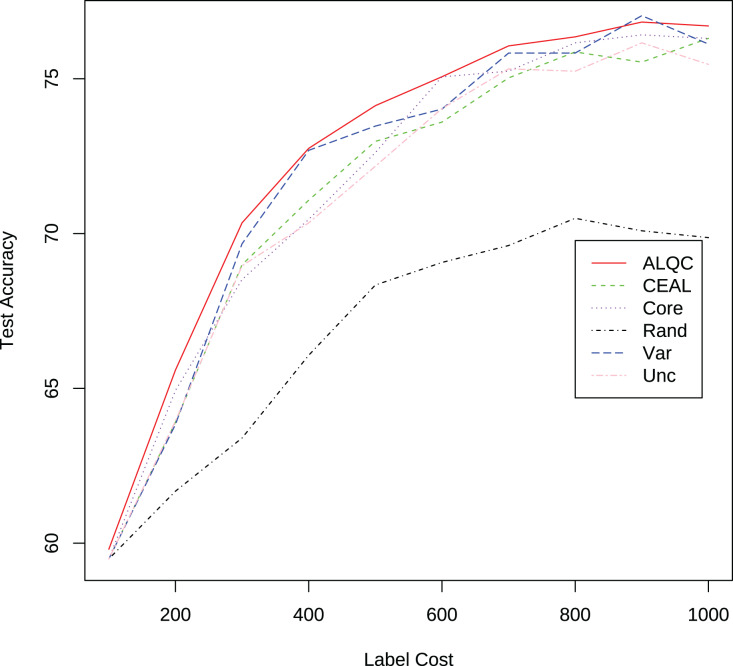
Experimental results on the AOI dataset.

There are a total of 3,100 samples in the AOI dataset, and all experiments start with 100 randomly selected labeled samples. In each iteration, 100 unlabeled samples are labeled, and 100 samples are removed from the unlabeled dataset. The experimental results are shown in Fig. 14 and Table 6. It can be seen that the proposed method outperforms other methods in this real application. In addition, the performance of the ALQC method steadily improves as the label cost increases, and its robust performance is more suitable for the needs of practical industrial applications.

**Table 6:** Test set accuracy of each methods on the AOI dataset.

Label cost	ALQC	CEAL	Core	Rand	Var	Unc
100	59.8050	59.4920	59.4920	59.4920	59.4920	59.4920
200	65.5930	63.9100	64.9340	61.6800	63.8380	63.9460
300	70.3480	68.9960	68.5180	63.3980	69.6720	68.9600
400	72.7530	71.0780	70.4580	66.0700	72.6890	70.3460
500	74.1310	72.9780	72.6140	68.3380	73.4720	72.1740
600	75.0650	73.6000	75.0620	69.0680	74.0220	74.0380
700	76.0620	75.0280	75.2460	69.6140	75.8300	75.3200
800	76.3540	75.8660	76.1600	70.4940	75.8320	75.2440
900	76.8320	75.5380	76.4180	70.0900	77.0380	76.1600
1,000	76.7060	76.3060	76.3040	69.8700	76.1220	75.4660

## Conclusion

In this work, we propose a new active learning method. By using the same sample selection metric as label quality control, we adaptively allocate labeling resources to active learning and to label quality control, thus improving the utilization of labeling resources. We design a practical algorithm for this method and prove that the algorithm only labels multiple duplicate samples with low probability, thus reducing the waste of labeling resources. The algorithm achieves the best results on both the benchmark datasets and the industrial application dataset. In the future, we plan to apply it to more practical problems.

## Supplemental Information

10.7717/peerj-cs.1480/supp-1 Supplemental Information 1 Original code. Please read "readme.pdf" in this file.

10.7717/peerj-cs.1480/supp-2 Supplemental Information 2 Revised code.

10.7717/peerj-cs.1480/supp-3 Supplemental Information 3 Final merged code package.

## Additional Information and Declarations

### Data Availability

The following information was supplied regarding data availability: The MNIST dataset is available at: http://yann.lecun.com/exdb/mnist/. The CIFAR-10 dataset is available at https://www.cs.toronto.edu/~kriz/cifar.html.
