# Supplementary material for: Active learning with label quality control
Source: PeerJ Comput Sci. 2023 Sep 8;9:e1480. doi: 10.7717/peerj-cs.1480 (PMC10496030; doi:10.7717/peerj-cs.1480)
Supplement: Supplemental Information 1 — Please read "readme.pdf" in this file. [file peerj-cs-09-1480-s001.zip › code/readme.pdf]

Directory: Environment

1. All source code is running on python 3.6 under linux.
2. You can use `pip install -r requirements.txt` to install the required packages.
3. You can also use `Dockerfile` to build a base environment and then use pip to install the required packages.

Directory: ActiveLearning_MNIST

1. Figure 3, Figure 4 ,Figure 5 and Figure 8 show the results from this code.
2. Usage: python3 main.py arg1 arg2 ..., the available arg are as follows:

    - --input_dir: Put the dataset and other configuration files in this directory.
    - --output_dir: Save logs and other results in this directory.
    - --tmp_dir: For temporary files.
    - --active_learning_method_name: All names in `Name2Method` at the end of `ActiveLearning.py`. The `LLB_L1CE` is our proposed method.
    - --init_indexes_name: File name for saving the index of randomly specified initial label samples. You can find some examples in `Data` directory.
    - --sequence_name: File name to limit the cumulative label cost per round. You can find some examples in `Data` directory.
    - --crowdsource_name: File name for recording the results of the simulated noise labeling results. You can find some examples in `Data` directory.
    - --crowdsource_accuracy: Specify the accuracy of simulated labeling.
    - --max_label_count: Specify the maximum number of times a single sample can be labeled.
    - --train_with_pseudo_split_number: In the paper, the value of k in K-fold.

3. Examples for Figure 3, Figure 4 and Figure 5.

    - CMD: `python3 main.py --active_learning_method_name=uncertainty --init_indexes_name=init/8f063b --`

`sequence_name=sequence_100_1000_100.json --crowdsource_name=8f063b_low_quality.json --crowdsource_accuracy=1 --max_label_count=1`

  - CMD: `python3 main.py --active_learning_method_name=LLB_L1CE --init_indexes_name=init/8f063b --sequence_name=sequence_100_1000_100.json --crowdsource_accuracy=0.95 --max_label_count=5`
  - CMD: `python3 main.py --active_learning_method_name=cleanlab_uncertainty_10 --init_indexes_name=init/8f063b --sequence_name=sequence_100_1000_100.json --crowdsource_accuracy=0.95 --max_label_count=5`
  - The `crowdsource_name` is from `Data/crowdsource/mnist` directory. `*_low_quality.json` are for Figure 3, and `*_high_quality.json` are for Figure 4.
  - Our proposed method `LLB_L1CE` and `cleanlab_uncertainty_*` don't need `crowdsource_name`. They decide by itself whether to check the labels of the labeled samples.

4. Examples for Figure 8.

  - CMD: `python3 main.py --active_learning_method_name=LLB_L1CE --init_indexes_name=init/8f063b --sequence_name=sequence_100_1000_100.json --crowdsource_accuracy=1 --max_label_count=1`

Directory: Fig_6

1. Figure 6 show the results from this code.
2. `Fig_6/run.sh` can start the test, you may first download MNIST dataset and copy init_index from `Data/init_indexes/mnist`.
3. Codes in `Fig_6` are a fork of `ActiveLearning_MNIST`.

Directory: Fig_7_1, Fig_7_2

1. Figure 7 show the results from these codes.
2. `Fig_7_1/run.sh` and `Fig_7_2/run.sh` can start the test, you may first download MNIST dataset.
3. Codes in `Fig_7_1` and `Fig_7_2` are a fork of `ActiveLearning_MNIST`.

Directory: ActiveLearning_CIFAR10

1. Figure show the results from this code.
2. Used in the same way as `ActiveLearning_MNIST` directory.
3. Examples for Figure 9 and Figure 10.

    - CMD: `python3 main.py --active_learning_method_name=core_set --init_indexes_name=init/39735b --sequence_name=sequence_1000_10000_1000.json --crowdsource_accuracy=1 --max_label_count=1`
    - CMD: `python3 main.py --active_learning_method_name=uncertainty_pseudo --init_indexes_name=init/39735b --sequence_name=sequence_1000_10000_1000.json --crowdsource_accuracy=1 --max_label_count=1`
    - The `init/39735b` and `sequence_1000_10000_1000.json` is from `Data` directory. You may first download CIFAT-10 dataset.

Directory: Data

1. `crowdsource`: Examples for arg `crowdsource_name`.
2. `init_indexes`: Examples for arg `init_indexes_name`.
3. `sequence_begin_end_step.json`: Examples for arg `sequence_name`.
4. Other public data

    - `resnet18-5c106cde.pth`: Pre-trained model provided by pytorch for initializing the model for the `Active Learning_CIFAR10` test. It can be download from `https://download.pytorch.org/models/resnet18-5c106cde.pth`.
    - MNIST and CIFAR-10 dataset: It can be download by `torchvision` package.

Data and code not available: Fig_13

1. The dataset used in Figure 13 is from a commercial company, just like the example shown in Figure 11. These samples would reveal trade secrets such as the company's product content and quality levels, so the

dataset is not available.

2. The AOI algorithm used for the experiments in Figure 13 is private algorithm of a commercial company. This algorithm is commercially important and therefore the code is not available.
3. The active learning algorithms used for the experiments in Figure 13 is just like these in `ActiveLearning_MNIST/ActiveLearning.py`.
4. Although we unfortunately cannot provide the complete algorithm and the full dataset in Figure 13, the other experiments with available codes and datasets in the paper are sufficient to verify the superiority of our proposed method. Figure 13 is only meant to show that our proposed method can be applied in real industrial tasks.
